# Potassium application enhances drought tolerance in sesame by mitigating oxidative damage and regulating osmotic adjustment

**DOI:** 10.3389/fpls.2022.1096606

**Published:** 2022-12-12

**Authors:** Sheng Fang, Huiyi Yang, Guangwei Wei, Tinghai Shen, Zehua Wan, Min Wang, Xiaohui Wang, Ziming Wu

**Affiliations:** Key Laboratory of Crop Physiology, Ecology, and Genetic Breeding, Ministry of Education/College of Agronomy, Jiangxi Agricultural University, Nanchang, China

**Keywords:** sesame, drought stress, potassium, reactive oxygen species, antioxidant, osmoregulation

## Abstract

Potassium (K) is known for alleviating the negative effects of abiotic stresses on plants. To explore the functions of K in controlling reactive oxygen species (ROS), antioxidant activities, and osmoregulation in sesame under drought stress, a pot experiment was conducted with three K levels (0, 60, and 120 kg ha^–1^, recorded as K0, K1, and K2, respectively) and exposed to well-watered (WW, 75% ± 5% soil relative water content) and drought-stressed (DS, 50% ± 5% soil relative water content) conditions. The results showed that DS stimulated the production of ROS such as increased hydrogen peroxide (H_2_O_2_), leading to lipid peroxidation as characterized by higher malondialdehyde (MDA) and, consequently, resulting in the decline in relative water content (RWC) and photosynthetic pigments as compared with WW plants. These adverse effects were exacerbated when drought stress was prolonged. Concurrently, K application alleviated the magnitude of decline in the RWC, chlorophyll *a*, and chlorophyll *b*, and plants applied with K exhibited superior growth, with the optimal mitigation observed under K2 treatment. Additionally, DS plants treated with K exhibited lower lipid peroxidation, higher antioxidant activities, and increased osmotic solute accumulation in comparison with plants under K deficiency, which suggested that exogenous K application mitigated the oxidative damages and this was more prominent under K2 treatment. Noteworthily, proline and soluble protein, respectively, dominated in the osmotic regulation at 3 and 6 days of drought stress according to the analysis of the quantitative comparison among different osmotically active solutes. Based on the correlation of the aforementioned traits and the analysis of variance on the interaction effects of drought stress and potassium, we propose that superoxide dismutase (SOD), glutathione reductase (GR), and MDA could be critical indicators in balancing ROS detoxification and reproduction. In summary, our studies suggest that optimized K application keeps a balance between the production of antioxidants and ROS and simultaneously affects osmoregulation to alleviate the damage from drought stress.

## Introduction

Sesame (*Sesamum indicum* L.) is an important high-quality oil crop with a long history of cultivation. However, due to the influence of climate change, more than 30% of the world’s arable land is facing the problem of insufficient water supply (Hoffmann and Sgrò, 2011; Thornton et al., 2014). As the most important abiotic stress, drought stress occurs frequently and severely, which threatens sesame production and sustainability in arid and semiarid regions (Pathak et al., 2014; Dossa et al., 2016; Baghery et al., 2022). The severity of water deficiency not only relies on the duration but also on the growth stage, i.e., seedling, vegetative, or reproductive stage, all of which have differential responses but ultimately all lead to yield loss. Particularly, drought stress burst at the flowering period is devastating, leading to reproductive failure, and consequently, results in shrunk yields (Zahoor et al., 2017c; Ul-Allah et al., 2020), since the recovery capability of sesame growth and development after rewatering at the flowering stage is limited.

Reactive oxygen species (ROS) are metabolic substances that may be produced by electron transfer in chloroplasts, mitochondria, and plasma membranes during plant metabolism, mainly including superoxide anion ( ${\text{O}}_{2}^{\mathrm{-1}}$ ), hydroxyl free radicals (OH^−1^), singlet oxygen (^1^O_2_), and hydrogen peroxide (H_2_O_2_). Generally, the production and elimination of ROS in plants maintain a dynamic balance but provoke a large amount of accumulation in response to drought stress (Xiong et al., 2018; Elsalahy et al., 2020), which exceeds the capacity of the scavenging system, leading to cell membrane lipid peroxidation or membrane lipid degreasing and, finally, forming the final product of the plant cell membrane lipid peroxidation—malondialdehyde (MDA). To counter the damages from ROS accumulation as well as protect macromolecules from oxidative damage, plants have accordingly developed a robust antioxidant system comprised of enzymes like catalase (CAT), peroxidase (POD), superoxide dismutase (SOD), ascorbate peroxidase (APX), and glutathione reductase (GR) (Choudhury et al., 2017; Zandalinas et al., 2018) and non-enzymatic compounds such as ascorbic acid (AsA), glutathione (GSH), and carotenoids, which scavenge ROS (Hasanuzzaman et al., 2020). For example, SOD can catalyze the disproportionation reaction of ( $\cdot {\text{O}}_{2}^{-}$ ) to form O_2_ and H_2_O_2_, and the H_2_O_2_, which is still toxic to cells at high concentrations, is then cleared by POD and CAT (Dietz et al., 2016). In addition, severe water stress affects plant growth and induces a range of osmotically active molecules/ions accumulated in plant cells including soluble sugars, proline, organic acids, potassium, etc. Among these compatible solutes, proline plays a major role in osmotic adjustment and also protects the cells by scavenging ROS (Dossa et al., 2017) with increased intensity of drought stress.

The availability of plant nutrients is crucial in drought stress adaptation and avoidance. Potassium (K), known as a stress alleviator plant nutrient, controls water losses from the plant (Farahani et al., 2019), delays leaf chlorosis and senescence (Weng et al., 2007), and mitigates the adverse consequences of drought stress by regulating the physio-biochemical characteristics (Hafsi et al., 2014) such as activating enzymes, osmoregulation, and membrane transport in cotton (Zahoor et al., 2017a), corn (Matłok et al., 2022), rice (Weng et al., 2007), and oilseed (Farahani et al., 2019). Drought increases ROS production by chloroplasts, peroxisomes, and mitochondria, and this could be further enhanced in K-deficient plants (Kaushal and Wani, 2016). Recent studies indicated that K is regarded not only as a pivotal nutrient but also as a signal transduction medium similar to ROS (Shabala, 2017), and conversely, ROS affects the K^+^ transporter across the cytomembrane (Anschütz et al., 2014). Under drought stress, exogenous K fertilizer increased the relative water content and reduced membrane damage, especially the enzymatic antioxidants produced for self-defense as evidenced by the higher activities of SOD, POD, CAT, GR, and APX and the lower content of MDA (Aksu and Altay, 2020; Siddiqui et al., 2021). Additionally, plants actively accumulate inorganic ions such as Na^+^, K^+^, and Ca^2+^ and organic substances like proline, soluble sugar, and soluble protein to reduce osmotic potential and maintain cell turgidity to mitigate damage caused by drought stress. However, the contribution of osmoregulation substances to osmotic adjustment varied with the duration of drought stress and crop species (Hessini et al., 2009; Hummel et al., 2010; Zhao et al., 2019). Exogenous application of potassium to drought-stressed plants induced higher contents of soluble sugar, proline, and amino acid (Zhao et al., 2019). Similarly, a higher proline content was detected in the leaves of drought-tolerant sesame because a large amount of proline accumulation in plants helps maintain osmotic balance and stabilize cell membranes, thus preventing electrolyte leakage and regulating ROS concentration within the normal level (Pourghasemian et al., 2020).

Traditional breeding, modern genetic tools, and advanced agronomical practices have been applied to mitigate yield loss resulting from drought stress (Sharma et al., 2019). Potassium application, an effective strategy for improving drought resistance by regulating osmotic and turgor pressure, is suggested to be used in agricultural production (Wang et al., 2013). Drought stress interrupts K diffusion in the soil toward the roots and, consequently, restricts K absorption. Normally, the supply of exogenous potassium is the most effective and direct pattern for plants to obtain K^+^. Numerous studies have been carried out to investigate the effects of water deficit and K nutrition on various field crops (Weng et al., 2007; Zahoor et al., 2017a; Farahani et al., 2019; Matłok et al., 2022), but how K protects sesame plants from the deleterious effects of drought stress is not well documented. Reports that addressed the interactive effects of potassium and drought stress regarding the generation of ROS, osmoregulation, and enzyme activities are lacking. Thus, we studied the responses of K application to antioxidant activities and osmotic adjustment substances of sesame under drought stress during the flowering stage. The objective of this study was to explore the physio-biochemistry mechanism of K application to improve sesame’s drought tolerance from the perspective of ROS metabolism and osmoregulation, which are expected to provide operative nutrient management strategies and guidance for alleviating the injurious effects of drought stress.

## Materials and methods

### Experimental design

The pot experiment was carried out in the Science and Technology Experiment Station of Jiangxi Agricultural University with a polythene shelter to avoid rainfall in June 2021. The soil was collected from the topsoil layer of the experimental site at 0–30 cm depth which contains 13.5 g kg^−1^ of organic matter, 0.85 g kg^−1^ of total nitrogen (N), 74.3 mg kg^−1^ of alkali hydrolyzed N, 19.6 mg kg^−1^ of available phosphorus (P), and 89.3 mg kg^−1^ of available potassium (K). The fertilizers phosphorus (superphosphate, 12% P_2_O_5_) and nitrogen (urea, 46% N) were applied at 90 kg ha^−1^ P_2_O_5_ and 120 kg ha^−1^ N as a basal dose. Healthy and plump sesame seeds were selected and sowed into a plastic pot (20 cm in diameter and 28 cm in height). After 2 weeks, the sesame seedlings were thinned to a single plant per pot, and every pot was considered a replication.

A randomized block design with three levels of potassium application and two water regime treatments was arranged in this experiment. The potassium treatment includes (0, 60, and 120 kg ha^−1^) K_2_O which was recorded as K0, K1, and K2, respectively, using potassium sulfate (K_2_SO_4_) as fertilizer. All plants were watered up to flower initiation, and then in one-half of the pots, watering was stopped for 6 days with a moisture level maintained at 50% ± 5% soil relative water content (SRWC), while the other half of the pots continued to be watered [well-watered (WW), 75% ± 5% SRWC]. Soil samples at 0–20 cm depth were collected daily from different pots during drought stress treatment at 6:00 p.m. local time with an auger to determine and hold soil moisture levels. The fresh weight of the soil samples was determined, followed by oven-drying at 105°C for 8 h. All physiological and biochemical indexes were measured at 3-day intervals once drought stress was imposed. The fourth fully expanded leaves from the apex of the sesame were sampled at 10:00 a.m. and then frozen in liquid nitrogen before being stored at −80°C for further analysis.

### Leaf relative water content and photosynthetic pigments

Relative water content (RWC) was determined with leaf fresh weight (FW), turgid weight (TW), and dry weight (DW) according to the following formula: RWC (%) = [(FW − DW)/(TW − DW)] × 100, where turgid weight was measured after the leaf has been incubated in distilled water for 6 h at laboratory room temperature.

Samples of 0.3 g from the fully expanded fourth leaves were ground in 5 ml of acetone (80%), and the extract was centrifuged at 10,000 rpm for 5 min. The absorbance (*A*) was recorded at wavelengths of 649 and 665 nm for chlorophyll assay by a V-5000 spectrophotometer. The pigments were calculated using the following formulas according to Sinaki et al. (2019):


$$\text{Chlorophyll }a\;{\text{(mg g}}^{-1}\text{) }=\;(13.95\times \text{A}665)\;-\;(6.88\times \text{A}649)$$

$$\text{Chlorophyll }b{\text{ (mg g}}^{-1}\text{) }=\;(24.96\times \text{A}649)\;-\;(7.32\times \text{A}665)$$

### Osmoregulation substances

Samples (0.2 g DW) were extracted three times by 5 ml of 80% (v/v) ethanol and incubated for 30 min at 80°C. Then, the mixture was centrifuged at 12,000×*g* for 5 min, and supernatants were collected and diluted to 25 ml with 80% (v/v) ethanol for soluble carbohydrate quantitation with an anthrone reagent at 620 nm (Hendrix, 1993). Moreover, free amino acid was measured using a ninhydrin reagent at 570 nm according to Fang et al. (2018).

To determine the proline content, 0.2 g of fresh sesame leaf was mixed in 10 ml of 3% (w/v) sulfosalicylic acid and centrifuged at 15,000×*g* for 10 min. Then, 2 ml of the supernatant was reacted with 2 ml of glacial acetic acid and 2 ml of acid ninhydrin in tubes in a boiling water bath for 1 h. Finally, 4 ml of toluene was applied to extract the mixture, and the absorbance of the aqueous phase was read at 520 nm (Pourghasemian et al., 2020). The soluble protein content was measured according to Bradford (1976) by using bovine serum albumin as a standard.

Variations of the osmotically active solute concentrations in sesame leaf were demonstrated by the coefficient of stress according to Yang et al. (2016), which was calculated as (*C*_DS-K(_ *_i_*_)_ − *C*_WW-K0_)/*C*_DS-K(_ *_i_*_)_ × 100%, where *C*, DS, WW, and *i* represent the concentration of osmotically active solutes, drought stress treatment, well-watered treatment, and three potassium rates, respectively.

### Determination of MDA, AsA, and H_2_O_2_

Lipid peroxidation was determined according to Hu et al. (2016a) with modifications. Fresh leaf (0.5 g) was ground into a homogenate with 5 ml of 10% trichloroacetic acid (TCA) and then centrifuged at 12,000×*g* for 10 min. An aliquot of 2 ml of supernatant was transferred to a test tube with 4 ml of 0.6% thiobarbituric acid (TBA) and mixed vigorously. The mixture was heated at 100°C for 15 min and cooled on ice to terminate the reaction. The MDA concentration was calculated from the absorbance at 450, 532, and 600 nm, respectively. Reduced AsA was extracted with 5 ml of 5% phosphoric acid, reacted with a mixture containing 3.6 mM of EDTA, 2 mM of dithiothreitol (DTT), 100 mM of KH_2_PO_4_, and 0.5% N-ethylmaleimide, and finally read at 525 nm.

H_2_O_2_ was extracted according to Okuda et al. (1991). The reaction mixture included 1 ml extraction, 400 μl of 12.5 mM DMAB in 0.375 M PBS (pH 6.5), 80 μl of MBTH, and 20 μl of peroxidase. The reaction was started by peroxidase at 25°C, and the rate of change in wavelength at 590 nm was recorded.

### Antioxidant enzyme extraction and analysis

A fresh leaf sample (0.3 g) was homogenized in 5 ml of 50 mM of phosphate buffer (pH 7.0) and 10 g L^−1^ of polyvinylpyrrolidone and centrifuged at 15,000×*g* for 20 min, and then the supernatants were collected as a crude extraction for enzyme activity determinations. 1) SOD (EC 1.15.1.1) activity was determined by monitoring the inhibition of photochemical reduction of Nitro blue tetrazolium (NBT) according to Tavakol et al. (2021). 2) CAT (EC 1.11.1.6) activity was measured by the ammonium molybdate method (Djanaguiraman et al., 2009). 3) POD (EC 1.11.1.7) activity was assayed by the change rate of H_2_O_2_ degradation in absorbance at 436 nm (Lin and Kao, 1999). 4) APX (EC 1.11.1.11) was evaluated by following the reduction at 290 nm with 3 ml of the reaction mixture which contains 50 mM of sodium phosphate buffer (pH 7.0), 0.1 mM of EDTA, 2.5 mM of H_2_O_2_, 0.1 mM of sodium ascorbate, and 200 μl of enzyme extract for 1 min (Nakano and Asada, 1981). 5) GR (EC 1.6.4.2) activity was determined by monitoring the extinction coefficient of NADPH at 340 nm (Lei et al., 2006).

### Data analysis

Analysis of variance was assessed by SPSS 17.0 statistics package, and a two-way ANOVA was also performed to evaluate the level of significance of K, drought stress, and their interactive effects. Differences at the *P*<0.05 level are considered statistically significant using the least significant difference (LSD) and are shown by different letters above the numbers of bars. Origin 2018 was applied for data processing and drawing of figures.

## Results

### Changes in phenotype, relative water content, and photosynthetic pigments

**Figure 1:**
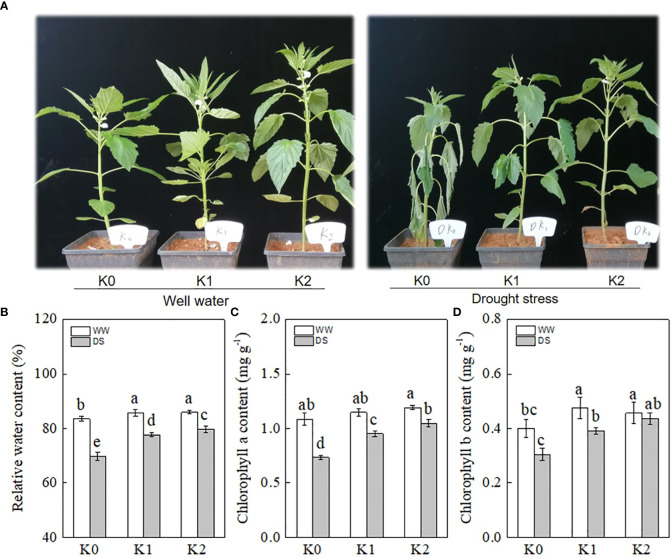
Effects of drought stress on phenotypic performance **(A)**, relative water content **(B)**, chlorophyll *a* **(C)**, and chlorophyll *b* **(D)** in sesame leaves at the flowering stage under different K levels. K0, K1, and K2 represent K application rates at 0, 60, and 120 kg ha^−1^ K_2_O, respectively. 3 d and 6 d indicate the sample date from the initiation of drought stress. Lowercase letters show significant differences at the *P*<0.05 level.

As shown in **Figure 1A**, the sesame plant without K supply exhibited a significant wilting phenotype following DS, whereas only a marginal damage symptom was observed in the K1 and K2 treatments. Additionally, WW plants with K application showed higher plant height and superior growth potential as compared with the K0 treatment.

The relative water content of the leaves exposed to potassium application was significantly increased as compared with K0 not only under DS but also under WW conditions (**Figure 1B**). DS plants showed an average reduction of 11.1% in RWC as compared with WW plants, and K application compensated for this reduction, with 9.6% and 12.0% of compensation observed under the K1 and K2 rates, respectively. The photosynthetic pigments were significantly decreased in DS plants in comparison with their non-DS counterparts. The chlorophyll *a* content was affected a little by the K rate in WW plants, whereas it significantly increased by 30.1% and 43.8% under K1 and K2 in DS plants as compared with K0 (**Figure 1C**). In parallel, K application reduced the chlorophyll *b* loss in DS plants (**Figure 1D**), and the lowest value was observed under K0 (0.30 mg g^−1^), followed by K1 (0.39 mg g^−1^) and K2 (0.44 mg g^−1^).

### Changes in osmotic adjustment substances

**Figure 2:**
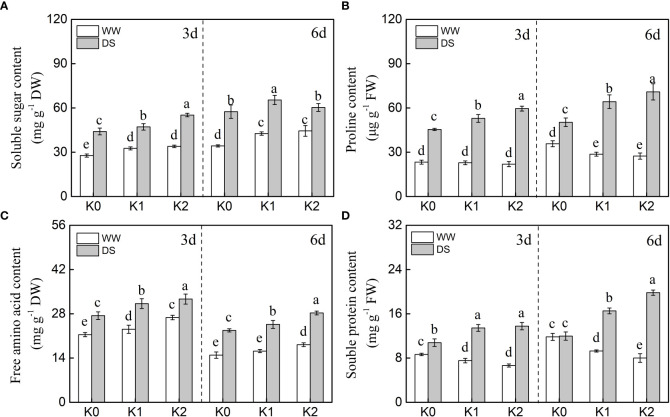
Effects of drought stress on osmotic adjustment substances [**(A)** soluble sugar, **(B)** proline, **(C)** free amino acid, **(D)** soluble protein) in sesame leaves at the flowering stage under different K levels. K0, K1, and K2 represent K application rates at 0, 60, and 120 kg ha^−1^ K_2_O, respectively. 3 d and 6 d indicate the sample date from the initiation of drought stress. Lowercase letters show significant differences at the *P*<0.05 level.

**Figure 3:**
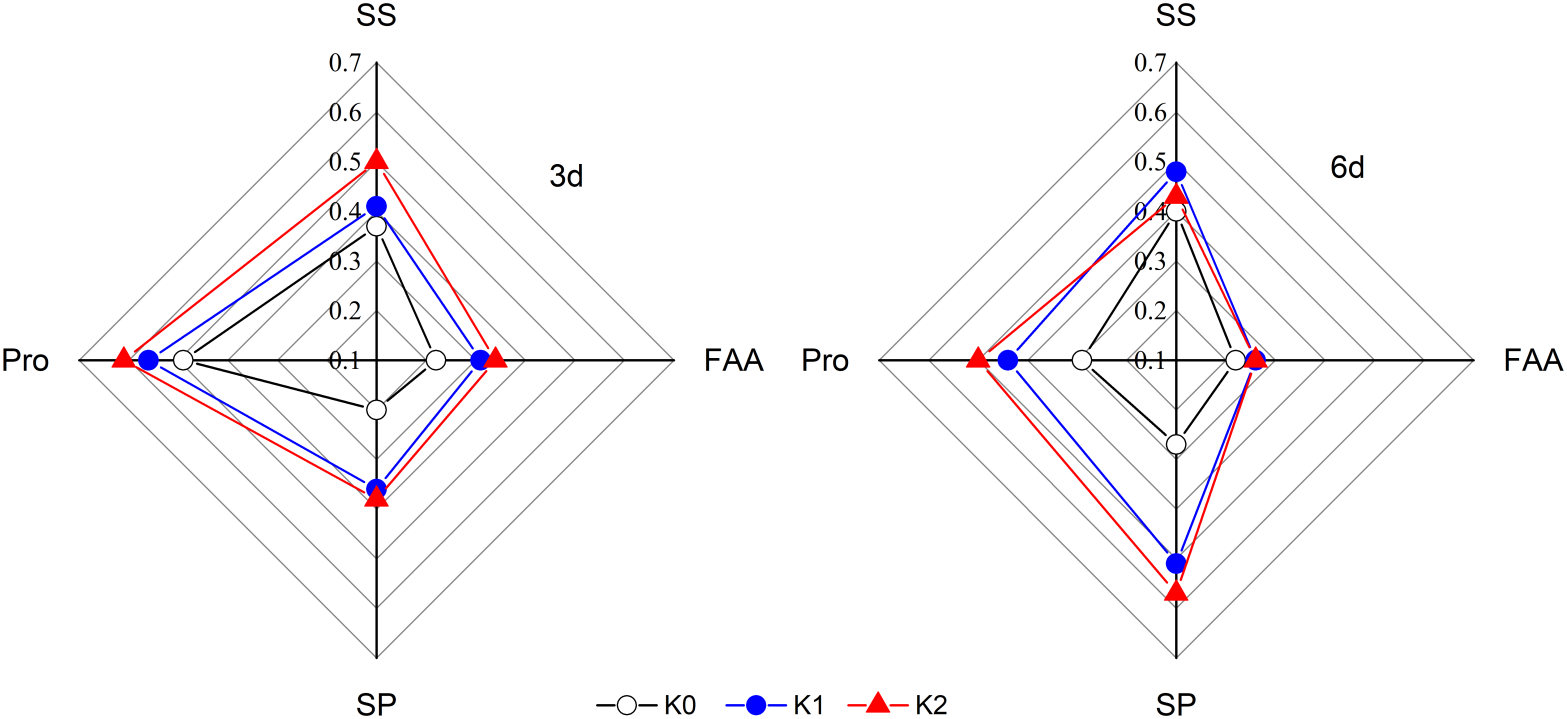
Variations of the osmotically active solute concentration in sesame leaf at 3 and 6 days, caused by drought stress, demonstrated by the coefficient of stress. K0, K1, and K2 represent K application rates at 0, 60, and 120 kg ha^−1^ K_2_O, respectively. SS, Pro, SP, and FAA stand for soluble sugar, proline, soluble protein, and free amino acid contents, respectively.

The osmotic adjustment substances including soluble sugar, proline, free amino acid, and soluble protein were markedly increased by 34.8%–52.5%, 107.1%–132.8%, 28.2%–52.7%, and 70.6%–75.2%, respectively, in DS plants as compared with WW plants, with the exception of soluble protein content detected at 6 days under K0 treatment (**Figure 2**). Additionally, a quantitative comparison between the contents of different osmotically active solutes in sesame leaf caused by drought stress (**Figure 3**) displayed that the impact of K application on proline, soluble sugar, and soluble protein was 1.90-, 1.08-, and 1.47-fold greater than that on free amino acid at 3 days of drought stress, respectively, whereas it was 1.66-, 1.82-, and 1.77-fold greater than that on amino acid at 6 days of drought stress.

The soluble sugar content (**Figure 2A**) was increased by 7.2%–13.7% and 4.8%–25.5% for the K1 and K2 treatments, respectively, as compared with K0 under drought stress, and similar trends were observed in WW plants, whereas K1 and K2 exhibited equal impacts at any sampling date. In addition, K application significantly enhanced the accumulation of free amino acid in drought-stressed (by 16.2%–16.5%) and well-watered (by 14.7%–16.6%) plants (**Figure 2C**), and this positive effect of K application was reinforced as the rate of K increased.

K supply decreased the proline and soluble protein contents as compared with K0 in the sesame leaves of WW plants, whereas a contrasting trend was demonstrated upon drought induction (**Figures 2B, D**). The enhanced accumulation rates of proline (average increased by 23.8% and 34.3%) and soluble protein (average increased by 25.9% and 52.5%) were exhibited under higher K levels in DS plants separately both 3 and 6 days of the sampling dates. Meanwhile, this positive promotion effect of K supply on proline and soluble protein was enhanced as K application increased for 6 days of drought stress. Noteworthily, the proline and soluble protein contents exhibited an opposite trend in WW plants, and a higher accumulation was shown in plants with K deficiency.

### Changes in MDA, H_2_O_2_, and AsA under drought stress and K application

**Figure 4:**
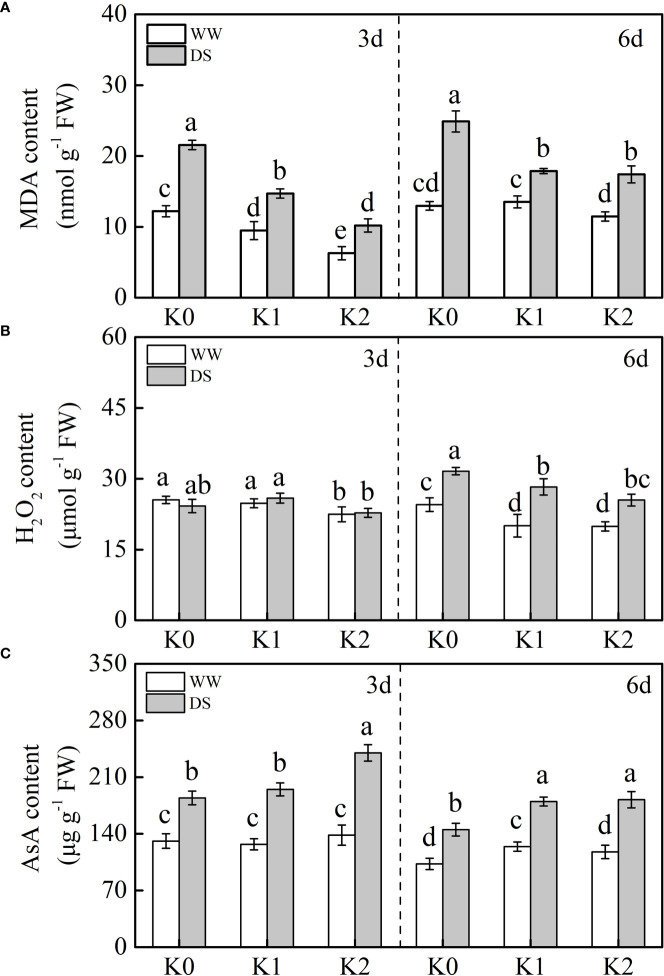
Effects of drought stress on MDA **(A)**, H_2_O_2_ **(B)**, and AsA **(C)** contents in sesame leaves at the flowering stage under different K levels. K0, K1, and K2 represent K application rates at 0, 60, and 120 kg ha^−1^ K_2_O, respectively. 3 d and 6 d indicate the sample date from the initiation of drought stress. Lowercase letters show significant differences at the *P*<0.05 level.

MDA is the final product of lipid peroxidation in the cell membrane. In our studies, MDA content was significantly increased by 58.7%–64.7% in DS plants, and this effect was more remarkable under 6 days of drought stress than under 3 days (**Figure 4A**). Specifically, K application reduced this increase to an extent, and the most prominent reduction in MDA content was demonstrated under K2 as compared with K0 under DS as well as WW conditions. There was no difference in H_2_O_2_ content between drought-stressed and well-watered plants regardless of K application rates at 3 days (**Figure 4B**). However, when plants were exposed to drought stress for 6 days, the H_2_O_2_ content was radically increased by 32.4% as compared with WW plants, and K supply mitigated the increasing trend partly with the least production of H_2_O_2_ observed under K2.

AsA exhibited a remarkable increase upon drought induction as compared with well-watered plants, whereas the increasing effects were suppressed as drought stress was prolonged (**Figure 4C**). K supply substantially enriched AsA content in sesame leaves under drought stress in comparison with plants without K supply at any sampling date. Additionally, no difference was found in the AsA content for 3 days regardless of K application under the WW condition, whereas it slightly increased by 20.8% under K1 at 6 days.

### Changes in antioxidant enzymes

SOD, CAT, POD, APX, and GR are the main enzymatic compounds involved in ROS. As shown in **Table 1**, compared with WW plants, drought induction increased SOD and CAT activities by 21.8% and 43.5% separately at 3 days, whereas prolonged drought stress (6 days) produced a greater increase in SOD and CAT activities, and this increase was enhanced with increasing rate of K application. In comparison with K0 under drought stress, the SOD activity increased by 11.1%–21.8% and 14.7%–30.1%, and CAT activity was increased by 12.4%–13.9% and 16.8%–41.6%, respectively, under K1 and K2 treatments. K application also increased SOD and POD activities in WW plants in comparison with K0, but there was no statistical difference between K1 and K2. During 3 days of drought stress, the POD activity in the leaves was reduced by 10.6% as compared with WW plants but inversely increased by 26.9% at 6 days. Noteworthily, the POD activity in drought stress was increased by 7.4% and 18.3%, respectively, under K1 and K2 at 6 days, whereas K application had a little effect on POD at 3 days.

**Table 1:** Effects of drought stress on the activities of SOD, POD, and CAT in sesame leaves at the flowering stage under different K levels.

		3 days (U g^−1^ FW)			6 days (U g^−1^ FW)		
Water regime	K rates	SOD	POD	CAT	SOD	POD	CAT
Well-watered	K0	118.5 c	254.8 b	198.9 d	133.9 e	358.1 d	157.1 d
	K1	128.5 c	258.4 b	261.0 c	157.9 d	367.0 d	202.6 c
	K2	131.7 bc	300.0 a	243.8 c	163.0 d	411.8 c	204.5 c
Drought stress	K0	141.8 b	232.5 b	305.3 b	205.8 c	443.3 bc	236.4 bc
	K1	157.6 a	245.4 b	347.9 a	250.6 b	476.2 b	265.6 b
	K2	161.9 a	258.5 b	356.7 a	267.7 a	524.3 a	334.8 a

The data are the means for three replications. Values followed by a different letter within the same column are significantly different at the P<0.05 level. K0, K1, and K2 represent K application rates at 0, 60, and 120 kg ha^−1^ K_2_O, respectively.

**Figure 5:**
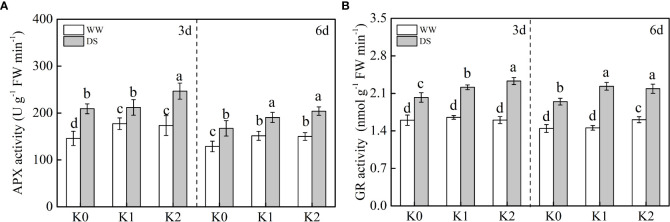
Effects of drought stress on the activities of APX **(A)** and GR **(B)** in sesame leaves at the flowering stage under different K levels. K0, K1, and K2 represent K application rates at 0, 60, and 120 kg ha^−1^ K_2_O, respectively. 3 d and 6 d indicate the sample date from the initiation of drought stress. Lowercase letters show significant differences at the *P*<0.05 level.

Compared with WW plants (**Figure 5**), APX and GR activities were synchronously enhanced under drought stress, with an increase of 30.6%–34.5% and 35.2%–41.2% observed, respectively. In our study, during drought stress, APX activity (**Figure 5A**) was only increased by 13.9% under K2 at 3 days, whereas it was comparatively increased by 17.9% and 21.9% under K1 and K2 at 6 days, respectively. GR activity exhibited a similar pattern to APX activity and increased with K application in drought-stressed plants, where specifically 9.4%–14.3% and 12.3%–15.3% of increases were detected under K1 and K2 in comparison with K0, respectively (**Figure 5B**). There was no statistical difference in APX activity between any given K treatments in WW plants at 3 days, but it increased by 11.8% under K2 in WW plants at 6 days.

### Correlation coefficients of ROS, osmoregulation, enzyme activity, and their variation sources

Analysis of variance (**Table 2**) exhibited statistically significant effects of drought stress and K application rate, respectively, on SOD, POD, CAT, APX, GR, AsA, MDA, and H_2_O_2_. However, the interaction effects of K and drought stress were only observed on the activities of SOD and GR and the content of MDA.

**Table 2:** Two-way ANOVA showing the significant effects of drought stress (DS), potassium (K), and their interactions on ROS metabolism-related traits in sesame leaf.

Factors	SOD	POD	CAT	APX	GR	AsA	MDA	H_2_O_2_
*F*_DS_	1,276.2**	80.4**	71.8**	75.9**	330.3**	205.8**	354.4**	86.3**
*F*_K_	118.9**	12.2**	15.4**	12.1**	12.8**	22.9**	46.1**	18.4**
*F*_DS_×_K_	14.4**	0.6^NS^	3.6^NS^	1.0^NS^	5.8*	2.9^NS^	34.4**	1.1^NS^

NS means non-significant; * and ** indicate significant differences at the P<0.05 and P<0.01 probability levels, respectively.

**Figure 6:**
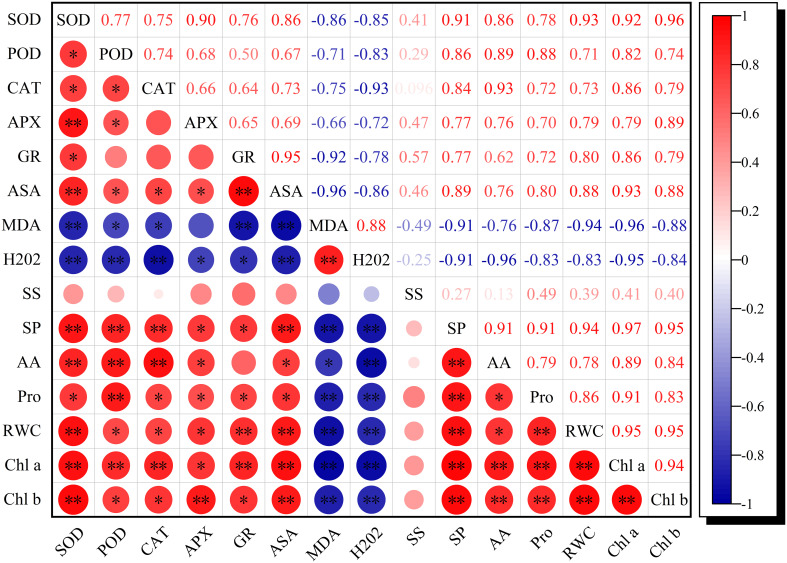
Correlation coefficients of RWC, Chl *a*, Chl *b*, ROS metabolism, and osmoregulation-related traits exposed to K and drought stress. *n* = 9, *R*_0.05_ = 0.6664, *R*_0.01_ = 00.7977. * and ** indicate significant differences at the *P*<0.05 and *P*<0.01 probability levels, respectively.

RWC, chlorophyll *a*, and chlorophyll *b* were synchronously significantly negatively correlated with MDA and H_2_O_2_, whereas they exhibited a significant positive correlation with the antioxidant enzyme activities and the osmoregulation-related index (except for soluble sugars), respectively (**Figure 6**), which implied that intensive antioxidant enzyme activities and osmotic adjustment substances were favorable for reducing oxidative stress, thus enhancing RWC and delaying senescence.

## Discussion

Drought stress occurs frequently all over the world. Although sesame is considered a moderately drought-tolerant crop, sustained and severe drought stress can ultimately reduce the production of sesame by limiting its growth and altering its physiological and biochemical activities. In the present study, drought stress significantly decreased the relative water content and photosynthetic pigments, whereas it increased the enzyme activities and peroxidation product of membrane lipids relevant to ROS metabolism as well as enhanced the accumulation of soluble sugar, proline, soluble protein, and free amino acids. K application improved the potential of the sesame plant to attenuate the adverse impacts of drought stress on leaf water relations, chlorophyll loss, ROS metabolism, and osmoregulation responding variably to different K supplies.

Drought-stressed plants showed wilting symptoms with the stems turning thinner and weaker and the leaves becoming soft and prolapsed, which were alleviated by K application (**Figure 1A**). Plant water relations are primarily determined by several physiological traits including leaf relative water content, which is an efficient factor to evaluate the continuous growth of crops under drought stress status (Farahani et al., 2020). In our studies, the RWC in DS plants was decreased by 11.1% as compared with WW plants, while K application alleviated this reduction (**Figure 1B**), with the lowest reduction (7.2%) observed under K2, which was consistent with the results of Aksu and Altay (2020) who showed that prolonged drought significantly reduced the RWC in the leaves and the application of potassium decreased the magnitude of decline. Consequently, drought-stressed plants grew better (**Figure 1A**) when exposed to K application as compared with K0 because of the positive effects of K on plant metabolism (Raza et al., 2013). Photosynthetic pigments such as chlorophyll *a* and chlorophyll *b* significantly affected energy production and light absorption, directly contributing to photosynthesis (Zahoor et al., 2017a). K plays a vital role in synthesizing the precursor of the chlorophyll pigment and improving the ability to convert radiant energy into chemical energy in chloroplasts (Jakli et al., 2017). DS significantly reduced chlorophyll *a* and *b* contents by 20.2% and 15.1% in our study (**Figures 1C, D**), respectively, which may result from enhanced pigment degradation or disrupted enzyme activities of the photosynthetic pigment (Saeidi et al., 2017). K supply improved chlorophyll content in DS and WW plants (**Figures 1C, D**), and the optimal result was observed under K2 treatment, which was partly compatible with the study of Aksu and Altay (2020) showing that chlorophyll *a* and chlorophyll *b* content improved remarkably with increasing levels of potassium.

Plants attempt to alleviate the damaging effects of drought stress on their cells through osmolyte accumulation such as soluble sugar (SS), free amino acid (FAA), soluble protein (SP), and proline (Pro) to acclimate to continuous drought stress (Dossa et al., 2017). Thus, these osmotically active solutes significantly increased, and subsequently, osmotic regulation occurs in cells during drought stress (**Figure 2**). Soil applied with K enhanced this accumulation in stressed plants to effectively keep plant functions at low leaf water status, and these motivating effects increased with increasing K rates. Those excessive osmolytes have been identified as vital contributors to osmotic adjustment exposed to water scarcity (Dossa et al., 2017; Zahoor et al., 2017b). Higher K rates exhibited less accumulation of osmolytes in the leaves in WW plants. Contrarily, K application significantly increased the accumulation of SS and FAA (**Figures 2A, C**) in well-watered plants as compared with K0 probably due to increased partitioning of stored carbon reserves (Wang et al., 2012) and increased SP degradation (Hu et al., 2016b) and, consequently, resulted in lower SP content under K1 and K2 treatments (**Figure 2D**). The SP, FAA, and Pro exhibited a significant positive correlation with the RWC, chlorophyll *a*, and chlorophyll *b* (**Figure 6**), which suggested that increased osmolyte accumulation was favorable for reducing oxidative stress, thus enhancing RWC and delaying senescence (Farahani et al., 2020; Matłok et al., 2022). Researchers claimed that SS and Pro are the primary osmotic adjustment substances exposed to moderate drought stress, but the ability of osmoregulation was suppressed as drought stress was prolonged (Hessini et al., 2009). Additionally, Zhao et al. (2019) reported that organic acid is the critical osmotic-regulating substance in plants under drought stress. Therefore, under drought conditions, the contribution of solutes to osmoregulation varies with plant species and drought duration (Hummel et al., 2010). In the present study, a quantitative comparison between the contents of different osmotically active solutes in sesame leaves caused by drought stress was conducted (**Figure 3**), and it suggested that proline mainly participates in osmotic regulation at 3 days of drought stress whereas soluble protein played a dominant role at 6 days of drought stress. It is consistent with the research that SP and Pro, recognized as the main drought stress indicators, were enriched in plants to balance osmotic pressure and scavenge ROS (Rai et al., 2012).

Drought stress has been demonstrated to stimulate the accumulation of ROS such as H_2_O_2_ and ${\text{O}}_{2}^{-}$ , which damage the cell constituents, resulting in leaf chlorosis and senescence, and potassium application is critical in mitigating the damage caused by osmotic stress (Hasanuzzaman et al., 2018; Matłok et al., 2022). Consistent with these studies, the content of H_2_O_2_ was significantly reduced under K supply in DS and WW plants (**Figure 4B**). Normally, the production and elimination of ROS in plants maintain a dynamic balance, while excessive accumulation of ROS induced by drought stress leads to cell membrane lipid peroxidation or degradation. MDA, a marker for lipid peroxidation, was markedly higher in drought-stressed plants (**Figure 4A**), which is in conformance with the results of Aksu and Altay (2020), and the degree of membrane lipid peroxidation was aggravated as drought stress was prolonged from initially 3 to 6 days. Expectedly, the application of K helps to decrease MDA in stressed and non-stressed plants in comparison to those plants without K application (**Figure 4A**), and the optimum effects of mitigation were observed under K2. This probably resulted from decreased ROS generation or upregulated scavenging activity of ROS under higher K levels (Zahoor et al., 2017c; Aksu and Altay, 2020). Concurrently, enhanced ROS attributed to drought stress stimulates redox signaling in plants and evokes an enforced defense mechanism such as enzymatic antioxidant activities and non-enzymatic compounds (Choudhury et al., 2017). The most important antioxidants in plants are SOD, POD, CAT, APX, GR, and AsA (Zandalinas et al., 2018). These antioxidants were synchronously significantly negatively correlated with MDA and H_2_O_2_ in drought-stressed sesame under K supply (**Figure 6**), suggesting that exogenous application of potassium mitigated the oxidative damages *via* improving the activities of antioxidants (Zahoor et al., 2017c; Sinaki et al., 2019; Bahar et al., 2021). Compared with WW plants, drought induction for 6 days significantly increased SOD, POD, and CAT activities, and this increase was enhanced with an increasing rate of K application (**Table 1**), which indicated that K assists to alleviate stress under drought conditions (Zahoor et al., 2017c; Matłok et al., 2022). There was no statistical difference in SOD, POD, and CAT activities at 3 days between K1 and K2 treatments despite that K application increased sesame oxidation resistance under drought stress (**Table 1**). To eliminate ROS damage, SOD can catalyze the ${\text{O}}_{2}^{-}$ to form O_2_ and H_2_O_2_ *via* disproportionation, whereas the toxic H_2_O_2_ was subsequently scavenged by POD and CAT (Pourghasemian et al., 2020). Therefore, the content of H_2_O_2_ remained stable under 3 days of water stress, whereas it increased with prolonged drought stress (**Figure 4B**), probably because ROS could not be completely scavenged by POD and CAT under prolonged drought stress (Wang et al., 2016), which consequently resulted in membrane lipid peroxidation and photosynthetic pigment degradation indicated by lower chlorophyll content (**Figures 1C, D**).

The AsA–GSH cycle is an additional vital antioxidant defense mechanism to cope with H_2_O_2_ attack which is regulated by enzymatic compounds such as APX and GR and non-enzymatic compounds like AsA (Hernandez et al., 2012). Ascorbic acid is a strong antioxidant protecting plants against damage when exposed to drought stress. In the present study (**Figure 4C**), the lowest AsA content was observed in WW plants without K supply. When drought stress occurred, an increase in AsA content was observed and concomitantly enhanced with the increased K level, which is consistent with the result of Aksu and Altay (2020). Compared with WW plants (**Figure 5**), APX and GR activities were synchronously enhanced under drought stress, and substantially higher values were detected under K2 in DS plants. Consequently, the reduction reaction of GSSG to GSH was promoted and might increase the AsA content produced *via* the reaction of GSH and dehydroascorbic acid to scavenge H_2_O_2_ (Hu et al., 2016a). Conclusively, the response of ROS accumulation and the corresponding existence of a robust antioxidant system induced by drought stress are complicated. Drought stress triggered ROS production by the mitochondria, chloroplasts, and peroxisomes, and this could be further enhanced in potassium-depleted plants (Kaushal and Wani, 2016). The analysis of variance (**Table 2**) exhibited statistically the remarkable interaction effect of two factors involving drought stress and K application rate on SOD, GR, and MDA, which implied that these might be the most pivotal indicators involved in the balance between ROS production and detoxification exposed to drought stress with K application.

## Conclusions

Optimal K application significantly alleviates ROS production and improves antioxidant activity under drought stress, which eventually results in the reduction of oxidative stress in the sesame plant, and this regulatory effect was more prominent with prolonged drought stress. Specifically, potassium application reduced the magnitude of decline in the relative water content and chlorophyll content induced by drought stress, and concurrently, osmotically active solutes and ascorbic acid were remarkably increased with K supply under drought stress. Plants with K application showed enhanced antioxidant activity (SOD, POD, CAT, APX, and GR) to detoxify the abundant ROS generated by drought stress and, consequently, alleviated the production of MDA and H_2_O_2_. Based on the analysis of variance on the interaction effects of DS and K, we deduced that SOD, GR, and MDA might be critical in balancing ROS detoxification and reproduction.

## Data availability statement

The original contributions presented in this study are included in the article/supplementary material, further inquiries can be directed to the corresponding author.

## Author contributions

SF and ZiW conceived and designed the experiment. HY, GW, TS, MW, XW, and Zew helped in conducting the experiment. SF and HY collected and statistically analyzed the data. SF wrote the draft. ZiW helped in improving the manuscript. All authors contributed to the article and approved the submitted version.

## References

[B1] Aksu G. Altay H. (2020). The effects of potassium applications on drought stress in sugar beet. Sugar Tech 22 (6), 1092–1102. doi: 10.1007/s12355-020-00851-w

[B2] Anschütz U. Becker D. Shabala S. (2014). Going beyond nutrition: regulation of potassium homoeostasis as a common denominator of plant adaptive responses to environment. J. Plant Physiol. 171 (9), 670–687. doi: 10.1016/j.jplph.2014.01.009 24635902

[B3] Baghery M. A. Kazemitabar S. K. Dehestani A. Mehrabanjoubani P. Naghizadeh M. M. Masoudi-Nejad A. (2022). Insight into gene regulatory networks involved in sesame (*Sesamum indicum* l.) drought response. Biologia 77 (4), 1181–1196. doi: 10.1007/s11756-022-01009-7

[B4] Bahar A. A. Faried H. N. Razzaq K. Ullah S. Akhtar G. Amin M.. (2021). Potassium-induced drought tolerance of potato by improving morpho-physiological and biochemical attributes. Agronomy 11 (12), 2573. doi: 10.3390/agronomy11122573

[B5] Choudhury F. K. Rivero R. M. Blumwald E. Mittler R. (2017). Reactive oxygen species, abiotic stress and stress combination. Plant J. 90 (5), 856–867. doi: 10.1111/tpj.13299 27801967

[B6] Dietz K.-J. Mittler R. Noctor G. (2016). Recent progress in understanding the role of reactive oxygen species in plant cell signaling. Plant Physiol. 171 (3), 1535–1539. doi: 10.1104/pp.16.00938 27385820 PMC4936595

[B7] Djanaguiraman M. Annie Sheeba J. Durga Devi D. Bangarusamy U. (2009). Cotton leaf senescence can be delayed by nitrophenolate spray through enhanced antioxidant defence system. J. Agron. Crop Sci. 195 (3), 213–224. doi: 10.1111/j.1439-037X.2009.00360.x

[B8] Dossa K. Wei X. Li D. Fonceka D. Zhang Y. Wang L.. (2016). Insight into the AP2/ERF transcription factor superfamily in sesame and expression profiling of DREB subfamily under drought stress. BMC Plant Biol. 16 (1), 171. doi: 10.1186/s12870-016-0859-4 27475988 PMC4967514

[B9] Dossa K. Yehouessi L. W. Likeng-Li-Ngue B. C. Diouf D. Liao B. Zhang X.. (2017). Comprehensive screening of some west and central African sesame genotypes for drought resistance probing by agromorphological, physiological, biochemical and seed quality traits. Agronomy 7 (4), 83. doi: 10.3390/agronomy7040083

[B10] Elsalahy H. H. Bellingrath-Kimura S. D. Roß C.-L. Kautz T. Döring T. F. (2020). Crop resilience to drought with and without response diversity. Front. Plant Sci. 11, 721. doi: 10.3389/fpls.2020.00721 32582251 PMC7283915

[B11] Fang S. Gao K. Hu W. Snider J. L. Wang S. Chen B.. (2018). Chemical priming of seed alters cotton floral bud differentiation by inducing changes in hormones, metabolites and gene expression. Plant Physiol. Biochem. 130, 633–640. doi: 10.1016/j.plaphy.2018.08.010 30130740

[B12] Farahani S. Majidi Heravan E. Shirani Rad A. H. Noormohammadi G. (2019). Effect of potassium sulfate on quantitative and qualitative characteristics of canola cultivars upon late-season drought stress conditions. J. Plant Nutr. 42 (13), 1543–1555. doi: 10.1080/01904167.2019.1628987

[B13] Farahani S. Shahsavari N. Mohammadi Arasteh M. (2020). Effect of potassium sulfate on the physiological characteristics of canola cultivars in late season drought stress conditions. J. Plant Nutr. 43 (9), 1217–1228. doi: 10.1080/01904167.2020.1727507

[B14] Hafsi C. Debez A. Abdelly C. (2014). Potassium deficiency in plants: effects and signaling cascades. Acta Physiol. Plant 36 (5), 1055–1070. doi: 10.1007/s11738-014-1491-2

[B15] Hasanuzzaman M. Bhuyan M. B. Nahar K. Hossain M. S. Mahmud J. A. Hossen M. S.. (2018). Potassium: a vital regulator of plant responses and tolerance to abiotic stresses. Agronomy 8 (3), 31. doi: 10.3390/agronomy8030031

[B16] Hasanuzzaman M. Bhuyan M. B. Zulfiqar F. Raza A. Mohsin S. M. Mahmud J. A.. (2020). Reactive oxygen species and antioxidant defense in plants under abiotic stress: Revisiting the crucial role of a universal defense regulator. Antioxidants 9 (8), 681. doi: 10.3390/antiox9080681 32751256 PMC7465626

[B17] Hendrix D. L. (1993). Rapid extraction and analysis of nonstructural carbohydrates in plant tissues. Crop Sci. 33 (6), 1306–1311. doi: 10.2135/cropsci1993.0011183X003300060037x

[B18] Hernandez M. Fernandez-Garcia N. Garcia-Garma J. Rubio-Asensio J. Rubio F. Olmos E. (2012). Potassium starvation induces oxidative stress in solanum lycopersicum l. roots. J. Plant Physiol. 169 (14), 1366–1374. doi: 10.1016/j.jplph.2012.05.015 22771251

[B19] Hessini K. Martínez J. P. Gandour M. Albouchi A. Soltani A. Abdelly C. (2009). Effect of water stress on growth, osmotic adjustment, cell wall elasticity and water-use efficiency in spartina alterniflora. Environ. Exp. Bot. 67 (2), 312–319. doi: 10.1016/j.envexpbot.2009.06.010

[B20] Hoffmann A. A. Sgrò C. M. (2011). Climate change and evolutionary adaptation. Nature 470 (7335), 479. doi: 10.1038/nature09670 21350480

[B21] Hu W. Lv X. Yang J. Chen B. Zhao W. Meng Y.. (2016a). Effects of potassium deficiency on antioxidant metabolism related to leaf senescence in cotton (*Gossypium hirsutum* l.). Field Crops Res. 191, 139–149. doi: 10.1016/j.fcr.2016.02.025

[B22] Hummel I. Pantin F. Sulpice R. Piques M. Rolland G. Dauzat M.. (2010). Arabidopsis plants acclimate to water deficit at low cost through changes of carbon usage: an integrated perspective using growth, metabolite, enzyme, and gene expression analysis. Plant Physiol. 154 (1), 357–372. doi: 10.1104/pp.110.157008 20631317 PMC2938159

[B23] Hu W. Zhao W. Yang J. Oosterhuis D. M. Loka D. A. Zhou Z. (2016b). Relationship between potassium fertilization and nitrogen metabolism in the leaf subtending the cotton (*Gossypium hirsutum* l.) boll during the boll development stage. Plant Physiol. Biochem. 101, 113. doi: 10.1016/j.plaphy.2016.01.019 26874296

[B24] Jakli B. Tavakol E. Trankner M. Senbayram M. Dittert K. (2017). Quantitative limitations to photosynthesis in K deficient sunflower and their implications on water-use efficiency. J. Plant Physiol. 209, 20–30. doi: 10.1016/j.jplph.2016.11.010 28012363

[B25] Kaushal M. Wani S. P. (2016). Plant-growth-promoting rhizobacteria: drought stress alleviators to ameliorate crop production in drylands. Ann. Microbiol. 66 (1), 35–42. doi: 10.1007/s13213-015-1112-3

[B26] Lei Y. Yin C. Li C. (2006). Differences in some morphological, physiological, and biochemical responses to drought stress in two contrasting populations of populus przewalskii. Physiol. Plantarum 127 (2), 182–191. doi: 10.1111/j.1399-3054.2006.00638.x

[B27] Lin C. C. Kao C. H. (1999). NaCl Induced changes in ionically bound peroxidase activity in roots of rice seedlings. Plant Soil. 216 (1), 147–153. doi: 10.1023/A:1004714506156

[B28] Matłok N. Piechowiak T. Królikowski K. Balawejder M. (2022). Mechanism of reduction of drought-induced oxidative stress in maize plants by fertilizer seed coating. Agriculture 12 (5), 662. doi: 10.3390/agriculture12050662

[B29] Nakano Y. Asada K. (1981). Hydrogen peroxide is scavenged by ascorbate-specific peroxidase in spinach chloroplasts. Plant Cell Physiol. 22 (5), 867–880.

[B30] Okuda T. Matsuda Y. Yamanaka A. Sagisaka S. (1991). Abrupt increase in the level of hydrogen peroxide in leaves of winter wheat is caused by cold treatment. Plant Physiol. 97 (3), 1265–1267. doi: 10.1104/pp.97.3.1265 16668520 PMC1081153

[B31] Pathak N. Rai A. K. Kumari R. Thapa A. Bhat K. V. (2014). Sesame crop: an underexploited oilseed holds tremendous potential for enhanced food value. Agric. Sci. 5 (06), 519. doi: 10.4236/as.2014.56054

[B32] Pourghasemian N. Moradi R. Naghizadeh M. Landberg T. (2020). Mitigating drought stress in sesame by foliar application of salicylic acid, beeswax waste and licorice extract. Agr. Water Manage. 231, 105997. doi: 10.1016/j.agwat.2019.105997

[B33] Rai A. C. Singh M. Shah K. (2012). Effect of water withdrawal on formation of free radical, proline accumulation and activities of antioxidant enzymes in ZAT12-transformed transgenic tomato plants. Plant Physiol. Biochem. 61, 108–114. doi: 10.1016/j.plaphy.2012.09.010 23127521

[B34] Raza S. Farrukh Saleem M. Mustafa Shah G. Jamil M. Haider Khan I. (2013). Potassium applied under drought improves physiological and nutrient uptake performances of wheat (*Triticum aestivun* l.). J. Soil Sci. Plant Nutr. 13 (1), 175–185.

[B35] Saeidi M. Moradi F. Abdoli M. (2017). Impact of drought stress on yield, photosynthesis rate, and sugar alcohols contents in wheat after anthesis in semiarid region of Iran. Arid Land Res. Manage. 31 (2), 204–218. doi: 10.1080/15324982.2016.1260073

[B36] Shabala S. (2017). Signalling by potassium: another second messenger to add to the list? J. Exp. Bot. 68 (15), 4003–4007. doi: 10.1093/jxb/erx238 28922770 PMC5853517

[B37] Sharma S. Chen C. Khatri K. Rathore M. S. Pandey S. P. (2019). Gracilaria dura extract confers drought tolerance in wheat by modulating abscisic acid homeostasis. Plant Physiol. Biochem. 136, 143–154. doi: 10.1016/j.plaphy.2019.01.015 30684843

[B38] Siddiqui M. H. Khan M. N. Mukherjee S. Alamri S. Basahi R. A. Al-Amri A. A.. (2021). Hydrogen sulfide (H_2_S) and potassium (K+) synergistically induce drought stress tolerance through regulation of h+-ATPase activity, sugar metabolism, and antioxidative defense in tomato seedlings. Plant Cell Rep. 40 (8), 1543–1564. doi: 10.1007/s00299-021-02731-3 34142217

[B39] Sinaki J. M. Dehaghi M. A. Rezvan S. Damavandi A. Khorami A. M. (2019). Sesame (*Sesame indicum* l.) biochemical and physiological responses as affected by applying chemical, biological, and nano-fertilizers in field water stress conditions. J. Plant Nutr. 43 (3), 456–475. doi: 10.1080/01904167.2019.1683189

[B40] Tavakol E. Jákli B. Cakmak I. Dittert K. Senbayram M. (2021). Optimization of potassium supply under osmotic stress mitigates oxidative damage in barley. Plants 11 (1), 55. doi: 10.3390/plants11010055 35009058 PMC8747552

[B41] Thornton P. K. Ericksen P. J. Herrero M. Challinor A. J. (2014). Climate variability and vulnerability to climate change: a review. Global Change Biol. 20 (11), 3313–3328. doi: 10.1111/gcb.12581 PMC4258067 24668802

[B42] Ul-Allah S. Ijaz M. Nawaz A. Sattar A. Sher A. Naeem M.. (2020). Potassium application improves grain yield and alleviates drought susceptibility in diverse maize hybrids. Plants 9 (1), 75. doi: 10.3390/plants9010075 31936011 PMC7020434

[B43] Wang R. Gao M. Ji S. Wang S. Meng Y. Zhou Z. (2016). Carbon allocation, osmotic adjustment, antioxidant capacity and growth in cotton under long-term soil drought during flowering and boll-forming period. Plant Physiol. Biochem. 107, 137–146. doi: 10.1016/j.plaphy.2016.05.035 27288990

[B44] Wang N. Hua H. Eneji A. E. Li Z. Duan L. Tian X. (2012). Genotypic variations in photosynthetic and physiological adjustment to potassium deficiency in cotton (*Gossypium hirsutum*). J. Photochem. Photobiol. 110, 1–8. doi: 10.1016/j.jphotobiol.2012.02.002 22387141

[B45] Wang M. Zheng Q. Shen Q. Guo S. (2013). The critical role of potassium in plant stress response. Int. J. Mol. Sci. 14 (4), 7370–7390. doi: 10.3390/ijms14047370 23549270 PMC3645691

[B46] Weng X. Y. Zheng C. J. Xu H. X. Sun J. Y. (2007). Characteristics of photosynthesis and functions of the water–water cycle in rice (*Oryza sativa* l.) leaves in response to potassium deficiency. Physiol. Plantarum 131 (4), 614–621. doi: 10.1111/j.1399-3054.2007.00978.x 18251852

[B47] Xiong H. Yu J. Miao J. Li J. Zhang H. Wang X.. (2018). Natural variation in OsLG3 increases drought tolerance in rice by inducing ROS scavenging. Plant Physiol. 178 (1), 451–467. doi: 10.1104/pp.17.01492 30068540 PMC6130013

[B48] Yang J. Hu W. Zhao W. Chen B. Wang Y. Zhou Z.. (2016). Fruiting branch k+ level affects cotton fiber elongation through osmoregulation. Front. Plant Sci. 7, 13. doi: 10.3389/fpls.2016.00013 26834777 PMC4722289

[B49] Zahoor R. Dong H. Abid M. Zhao W. Wang Y. Zhou Z. (2017a). Potassium fertilizer improves drought stress alleviation potential in cotton by enhancing photosynthesis and carbohydrate metabolism. Environ. Exp. Bot. 137, 73–83. doi: 10.1016/j.envexpbot.2017.02.002

[B50] Zahoor R. Zhao W. Abid M. Dong H. Zhou Z. (2017b). Potassium application regulates nitrogen metabolism and osmotic adjustment in cotton (*Gossypium hirsutum* l.) functional leaf under drought stress. J. Plant Physiol. 215, 30–38. doi: 10.1016/j.jplph.2017.05.001 28527336

[B51] Zahoor R. Zhao W. Dong H. Snider J. L. Abid M. Iqbal B.. (2017c). Potassium improves photosynthetic tolerance to and recovery from episodic drought stress in functional leaves of cotton (*Gossypium hirsutum* l.). Plant Physiol. Biochem. 119, 21–32. doi: 10.1016/j.plaphy.2017.08.011 28843133

[B52] Zandalinas S. I. Mittler R. Balfagón D. Arbona V. Gómez-Cadenas A. (2018). Plant adaptations to the combination of drought and high temperatures. Physiol. Plantarum 162 (1), 2–12. doi: 10.1111/ppl.12540 28042678

[B53] Zhao W. Dong H. Zahoor R. Zhou Z. Snider J. L. Chen Y.. (2019). Ameliorative effects of potassium on drought-induced decreases in fiber length of cotton (*Gossypium hirsutum* l.) are associated with osmolyte dynamics during fiber development. Crop J. 7 (5), 619–634. doi: 10.1016/j.cj.2019.03.008

